# Screening for eating disorders and low energy availability in female trail runners: A cross-sectional study

**DOI:** 10.1371/journal.pone.0348896

**Published:** 2026-05-13

**Authors:** Carolyn Hill, Cécile Vigne, Patrick Basset, Volker Scheer, David Baud

**Affiliations:** 1 Materno-Fetal and Obstetric Research Unit, Obstetric Service, Woman-Mother-Child Department, University Hospital of Lausanne (CHUV) and University of Lausanne (UNIL), Lausanne, Switzerland; 2 Ultra Sports Science Foundation, Pierre-Bénite, France; Polytechnic University of Guarda, PORTUGAL

## Abstract

This cross-sectional study aims to screen for eating disorders and low energy availability, as well as identify associated factors among female trail runners. An online survey was used, collecting participants’ sociodemographic data, trail running habits, medical indicators, and screening questionnaires for eating disorders (BEDA-Q) and low energy availability (LEAF-Q). A total of 276 female trail runners were included in the analysis. The mean age was 36.2 (± 8.1) years and body mass index (BMI) was 22.0 (± 2.5) kg/m^2^. They reported 6.2 (± 5.0) years of trail running experience and 7.7 (± 4.3) hours of training per week. Statistical analysis was conducted to analyse the frequency of positive screening based on the sociodemographic characteristics, trail running habits, and medical indicators. Multivariate logistic regression was used to analyse factors associated with a positive screening for eating disorders and low energy availability. A total of 53.6% participants had a positive BEDA-Q score (defined as a score ≥ 0.27), screening positive for eating disorders. Independent risk factors associated with a positive screening for eating disorders were BMI above 24 kg/m^2^ (p = 0.001), high school education (p = 0.049), doctoral degree (p = 0.023) and a positive screening for depression (p < 0.001). Overall, 55.1% participants had a positive LEAF-Q score (defined as a score ≥ 8), indicating a risk of low energy availability. Associated factors were a decrease in libido (p = 0.043) and a positive screening for depression (p = 0.027). Trail running more than 3 years (p = 0.020) was the only independent risk factor for low energy availability. The independent protective factors for low energy availability were BMI above 24 kg/m2 (p = 0.047) and doctoral degree (p = 0.014). An association between eating disorders and low energy availability was also demonstrated. We present novel data on eating disorders and low energy availability in female trail runners. The elevated rates of these conditions highlight the importance of creating awareness, screening, and early interventions in this population.

## Introduction

Eating disorders (EDs) are mental health conditions, marked by a persistent disturbance in eating-related behaviors that significantly impair physical or psychological health [1]. The lifetime prevalence of EDs in the general population is reported to be 0.91%, rising to 2.58% among women [2]. Athletes represent a group at elevated risk for EDs, particularly those in endurance, aesthetic, and weight-dependent sports, where the belief that reduced body weight improves performance may contribute to this vulnerability [3,4]. Among female athletes, the prevalence of EDs is estimated to range from 6% to 45%, depending on the sport and the screening tool used [4–13]. These rates are three to five times higher than those observed in male athletes [9,10]. Given that EDs can lead to insufficient energy intake, whether through restrictive eating or compensatory behavior, athletes with EDs are particularly vulnerable to developing low energy availability [14–17].

Low energy availability (LEA) occurs when energy intake is insufficient to meet the demands of both training and the body’s physiological functions, such as growth, immunity, reproduction, and thermoregulation [16]. This energy imbalance can happen with or without an eating disorder, resulting in conscious or unconscious under-eating [4,16]. LEA is a significant health concern, as it can lead to Relative Energy Deficiency in Sport (RED-S) [17,18], a syndrome associated with serious health risks, such as impaired reproductive function, decreased bone density, weakened immune response, reduced protein synthesis, and negative cardiovascular effects [14,18]. RED-S equally affects athletes’ performance and can decrease endurance, training response, coordination and concentration, as well as, increase injury risk and impair judgment [17]. Furthermore, female athletes are particularly vulnerable to LEA compared to males, due to differences in endocrine and metabolic responses and heightened sensitivity to energy deficits [19,20]. According to existing research, the prevalence of LEA among female endurance athletes ranges from 18% to 80% [9,10,21–27], and a positive association between EDs and LEA has been widely demonstrated in this population [9,10,28]. However, the mechanisms underlying these associations may be more complex than energy deficiency alone. RED-S is widely used in sports medicine, but it is not a formally recognized clinical diagnosis, and concerns have been raised regarding its empirical basis [29]. Alternative models, such as the theory of allostatic load, suggest that cumulative physiological and psychosocial stress, rather than energy deficiency alone, may contribute to hormonal disturbances and mental health symptoms observed in female athletes [29].

In athlete cohorts, the assessment of EDs and LEA is often done by means of self-reported questionnaires. The Brief Eating Disorder in Athletes Questionnaires (BEDA-Q) is a screening tool for EDs and is included in the International Olympic Committee’s Sport Mental Health Assessment Tool 1 [30,31]. While the Low Energy Availability in Females Questionnaire (LEAF-Q) is used to identify female athletes with symptoms of LEA and who may be at risk of developing RED-S [21].

Trail running is an outdoor sport in which participants run in natural, off-road settings, with paved sections limited to 20% of the total route. Distances can range from a few kilometers to well over one hundred kilometers [32,33]. This sport has grown rapidly in popularity worldwide, with female participation increasing more than threefold between 1997 and 2022 [34]. The combination of prolonged physical effort, challenging environmental conditions, and demanding training inherent to this discipline may increase participants’ vulnerability to health concerns, such as EDs and LEA.

A study focusing on trail runners, found that 48% of female trail runners were at risk of EDs, compared to 27% of male trail runners [35]. In the same cohort, LEAF-Q results showed that 49.7% of female trail runners were at risk of LEA, with younger athletes more likely to be affected [35]. Additionally, a positive correlation was observed between the risk of EDs and LEA [35].

Female endurance athletes represent a high-risk population for EDs and LEA as they are more affected than males and practice a high energy expenditure sport, where leanness is viewed as an advantage for performance. In addition, the challenging conditions of trail running might increase this risk, making female trail runners at elevated risk for EDs and LEA. However, data specific to this population remain limited, making research on factors related to EDs and LEA essential for these athletes, their teams and health professionals. A better understanding would enable early detection and support effective psychological and nutritional interventions, helping to prevent impairments in health and performance [36,37]. This study therefore aimed to screen for EDs and LEA in female trail runners and identify factors associated with a positive screening for these conditions.

## Materials and methods

This cross-sectional study used an online survey conducted among female trail runners. The inclusion criteria were to be female, at least 18 years old and to regularly practice trail running (training at least once a week or competing). As part of the survey, written informed consent was obtained from all participants. The study was approved by the Comité Ético Investigacíon from University Hospital Elche, Spain, and was conducted in accordance with the Declaration of Helsinki [38].

The survey, delivered via Google Forms, was shared through Ultra Sports Science Foundation’s social media and remained active from October 2024 to March 2025. All data were self-reported, and participants had to answer each question to continue.

The questionnaire included information on participant’s characteristics, trail running habits and medical history. Trail running was defined as a foot race in a natural environment over a variety of different terrains with minimal paved roads, not exceeding 20–25% of the total race course [32]. Distance categories were defined as short trail (<42km), long trail (42–80 km) and ultra trail (>80km). Performance level included self-classification of elite and non-elite trail runner, with elite runners defined as professional, or collegiate, participating at the national or international level [31]. Total years trail running was separated at three years to distinguish between novice and experienced runners. Weekly training kilometers was categorized using a 50 km/week threshold, a benchmark for differentiating low from moderate-to-high mileage in trail runners [35] and a representative average training load for recreational female athletes [24].

The survey also contained three validated questionnaires, the Brief Eating Disorder in Athletes Questionnaire (BEDA-Q), the Low Energy Availability in Females Questionnaire (LEAF-Q), and the Patient Health Questionnaire-9 (PHQ-9), which, respectively, assess the risk of eating disorders, the risk of low energy availability and the risk of depression among participants.

The BEDA-Q [30] is a screening tool used to identify eating disorders in female athletes. This questionnaire comprises 9 questions assessing drive for thinness, body dissatisfaction, perfectionism and dieting. The score was calculated by dividing each participant’s raw score by the maximum possible score (19), resulting in values ranging from 0 to 1. Higher scores indicate a greater risk of an eating disorder, while lower scores indicate a lower risk. A BEDA-Q score ≥ 0.27 was the cutoff used to identify runners at risk of an eating disorder.

The LEAF-Q [21] is a screening instrument designed to identify female athletes at risk of low energy availability, and consequently, at risk of developing Relative Energy Deficiency in Sport (RED-S). It is comprised of 25 questions related to injuries, gastrointestinal function, menstrual function and the use of contraceptives. A scoring key allows us to calculate a total score ranging from 0 to 49, with a score ≥ 8 indicating a risk of low energy availability.

The PHQ-9 [39,40] is a validated diagnostic screening tool, used to assess the risk of depression among the participants. This brief questionnaire measures the severity of depressive symptoms and suicidal thoughts over the preceding 14 days. The total score ranges between 0–27 and scores ≥ 10 identifying major depression. The results of the PHQ-9 were used as a variable and compared with the outcomes of the BEDA-Q and the LEAF-Q.

Continuous variables were grouped into ordinal categories with roughly equal sample sizes, and questionnaire scores were dichotomized into positive and negative categories based on their respective validated cutoff values. A BMI threshold of 24 kg/m² was used as a cutoff to identify athletes approaching the overweight category, ensuring a clinically relevant subgroup while ensuring a sufficient sample size to maintain statistical power. Differences between groups were assessed with Pearson’s χ² tests. The effect size was calculated using the Cramér’s V and interpreted according to Cohen (1988) [41]: 0.10–0.29 weak, 0.30–0.49 moderate, and ≥ 0.50 strong association. Multivariate logistic regression was used to identify factors independently associated with positive questionnaire scores. All variables listed in Tables 1–3, except height and weight, were included as predictors in the models, and stepwise backward elimination was applied to retain only statistically significant predictors. To ensure model stability and reduce the risk of overfitting, we followed the Events-Per-Variable (EPV) criterion, targeting a ratio above the recommended 10 events per predictor [42]. All analyses were considered statistically significant at p < 0.05. Statistical analyses were performed using STATA-18 (Stata Corporation, College Station, USA).

**Table 1:** Socio-demographic characteristics – Pearson’s χ² test.

	Sample size	BEDA-Q ≥ 0.27			LEAF-Q ≥ 8		
		(at risk of having an eating disorder)			(at risk of low energy availability)		
	(N = 276)	(N = 148)	p value	Cramér’s V	(N = 152)	p value	Cramér’s V
**Age (years)**			0.875	0.03		0.242	0.10
≤ 30	72 (26.1%)	37 (51.4%)			44 (61.1%)		
31-40	123 (44.6%)	66 (53.7%)			61 (49.6%)		
> 40	81 (29.4%)	45 (55.6%)			47 (58.0%)		
**Weight (kg)**			0.004	0.20		0.188	0.11
≤ 55	81 (29.4%)	37 (45.7%)			44 (54.3%)		
56-64	117 (42.4%)	57 (48.7%)			71 (60.7%)		
> 64	78 (28.3%)	54 (69.2%)			37 (47.4%)		
**Height (cm)**			0.580	0.06		0.377	0.08
≤ 160	65 (23.6%)	37 (56.9%)			37 (56.9%)		
161-169	137 (49.6%)	75 (54.7%)			70 (51.1%)		
> 169	74 (26.8%)	36 (48.7%)			45 (60.8%)		
**BMI (kg/m2)**			0.001	0.19		0.142	0.09
≤ 24	218 (79.0%)	106 (48.6%)			125 (57.3%)		
> 24	58 (21.0%)	42 (72.4%)			27 (46.6%)		
**Nationality**			0.590	0.03		0.231	0.07
Europe	160 (58.0%)	88 (55.0%)			93 (58.1%)		
Non Europe	116 (42.0%)	60 (51.7%)			59 (50.9%)		
**Civil status**			0.814	0.06		0.424	0.10
Single	77 (27.9%)	38 (49.4%)			44 (57.1%)		
Married	97 (35.1%)	55 (56.7%)			54 (55.7%)		
Partnership	93 (33.7%)	50 (53.8%)			47 (50.5%)		
Divorced	9 (3.3%)	5 (55.6%)			7 (77.8%)		
**Education level**			0.065	0.16		0.061	0.16
High school	26 (9.4%)	18 (69.2%)			11 (42.3%)		
Bachelor	111 (40.2%)	60 (54.1%)			70 (63.1%)		
Master	109 (39.5%)	50 (45.9%)			59 (54.1%)		
Doctorate	30 (10.9%)	20 (66.7%)			12 (40.0%)		
**Employment status**			0.310	0.09		0.937	0.02
Employed	198 (71.7%)	110 (55.6%)			108 (54.6%)		
Self-employed	54 (19.6%)	24 (44.4%)			30 (55.6%)		
Unemployed	24 (8.7%)	14 (58.3%)			14 (58.3%)		
**Smoker**			0.619	0.03		0.486	0.04
No	236 (85.5%)	128 (54.2%)			132 (55.9%)		
Yes	40 (14.5%)	20 (50.0%)			20 (50.0%)		

**Table 2:** Trail running characteristics – Pearson’s χ² test.

	Sample size	BEDA-Q ≥ 0.27			LEAF-Q ≥ 8		
		(at risk of having an eating disorder)			(at risk of low energy availability)		
	(N = 276)	(N = 148)	p value	Cramér’s V	(N = 152)	p value	Cramér’s V
**Total years trail running**			0.659	0.03		0.053	0.12
≤ 3	103 (37.3%)	57 (55.3%)			49 (47.6%)		
> 3	173 (62.7%)	91 (52.6%)			103 (59.5%)		
**Performance level**			0.415	0.05		0.538	0.04
Elite	16 (5.8%)	7 (43.8%)			10 (62.5%)		
Non-Elite	260 (94.2%)	141 (54.2%)			142 (54.6%)		
**Trail running distance**			0.200	0.11		0.424	0.08
Short trail <42km	155 (56.2%)	76 (49.0%)			80 (51.6%)		
Long trail 42–80 km	86 (31.2%)	50 (58.1%)			51 (59.3%)		
Ultra trail >80km	35 (12.7%)	22 (62.9%)			21 (60.0%)		
**Weekly training (hours)**			0.889	0.03		0.320	0.09
0-5	92 (33.3%)	48 (52.2%)			48 (52.2%)		
6-9	107 (38.8%)	57 (53.3%)			56 (52.3%)		
≥ 10	77 (27.9%)	43 (55.8%)			48 (62.3%)		
**Weekly training (km)**			0.261	0.07		0.326	0.06
≤ 50	167 (60.5%)	85 (50.9%)			88 (52.7%)		
> 50	109 (39.5%)	63 (57.9%)			64 (58.7%)		
**Yearly race participation**			0.405	0.08		0.709	0.05
0-3	104 (37.7%)	53 (51.0%)			55 (52.9%)		
4-6	108 (39.1%)	56 (51.9%)			59 (54.6%)		
≥ 7	64 (23.2%)	39 (60.9%)			38 (59.4%)		

**Table 3:** Medical, Gynecological-Obstetrical characteristics and PHQ-9 score – Pearson’s χ² test.

	Sample size	BEDA-Q ≥ 0.27			LEAF-Q ≥ 8		
		(at risk of having an eating disorder)			(at risk of low energy availability)		
	(N = 276)	(N = 148)	p value	Cramér’s V	(N = 152)	p value	Cramér’s V
**Total past pregnancies**			0.406	0.05		0.241	0.07
Never	154 (55.8%)	86 (55.8%)			80 (52.0%)		
≥ 1	122 (44.2%)	62 (50.8%)			72 (59.0%)		
**Total past births**			0.636	0.03		0.532	0.04
Never	168 (60.9%)	92 (54.8%)			90 (53.6%)		
≥ 1	108 (39.1%)	56 (51.9%)			62 (57.4%)		
**Oral contraceptives**			0.894	0.01		0.939	0.01
No	232 (84.1%)	124 (53.5%)			128 (55.2%)		
Yes	44 (14.1%)	24 (54.6%)			24 (54.6%)		
**Libido during training**			0.548	0.07		0.043	0.15
It stays the same	145 (52.5%)	75 (51.7%)			70 (48.3%)		
It decreases	91 (33.0%)	53 (58.2%)			59 (64.8%)		
It increases	40 (14.5%)	20 (50.0%)			23 (57.5%)		
**History of urinary incontinence**			0.326	0.06		0.200	0.08
No	153 (55.4%)	78 (51.0%)			79 (51.6%)		
Yes	123 (44.6%)	70 (56.9%)			73 (59.4%)		
**Urinary incontinence during trail**			0.843	0.01		0.547	0.04
No	157 (56.9%)	85 (54.1%)			84 (53.5%)		
Yes	119 (43.1%)	63 (52.9%)			68 (57.1%)		
**History of pelvic prolapse**			0.354	0.06		0.816	0.01
No	264 (95.7%)	140 (53.0%)			145 (54.9%)		
Yes	12 (4.4%)	8 (66.7%)			7 (58.3%)		
**History of stress fractures**			0.470	0.04		0.597	0.03
No	237 (85.9%)	125 (52.7%)			129 (54.4%)		
Yes	39 (14.1%)	23 (59.0%)			23 (59.0%)		
**PHQ-9 score**			<0.001	0.28		0.027	0.13
< 10	222 (80.4%)	104 (46.9%)			115 (51.8%)		
≥ 10	54 (19.6%)	44 (81.5%)			37 (68.5%)		

## Results

Data included a total of 318 female trail runners, of which 42 were excluded because they were menopausal, as the validity of the LEAF-Q in menopausal women has not been established. Of the 276 female trail runners included in the analysis, the mean age was 36.2 (± 8.1) years and body mass index (BMI) was 22.0 (± 2.5) kg/m^2^. They reported 6.2 (± 5.0) years of trail running experience, 7.7 (± 4.3) hours of training per week, and participation in 5.0 (± 2.8) competitions per year. A total of 148 (53.6%) participants scored positive on the BEDA-Q (≥0.27), screening positive for eating disorders, and 152 (55.1%) participants had a positive LEAF-Q score (≥8), indicating a risk of low energy availability. Of the participants, 206 (74.6%) scored ≥8 on the LEAF-Q and/or ≥0.27 on the BEDA-Q, and 94 (34%) of them tested positive on both questionnaires.

The sociodemographic characteristics of trail runners with positive BEDA-Q and LEAF-Q scores are presented in Table 1. Body weight and BMI were the only variables which showed a statistically significant difference for the BEDA-Q score. Specifically, participants with a BMI above 24 kg/m^2^ had a greater risk of screening positive for eating disorders compared to those with a lower BMI (p = 0.001, Cramér’s V = 0.19), and runners with higher body weight were more likely to score positive on the BEDA-Q than those with lower weight (p = 0.004, Cramér’s V = 0.20).

Table 2 shows the participants’ trail running history. Performance level, trail running distance, weekly training hours and kilometers, and yearly race participation showed no significant differences in either the BEDA-Q score or the LEAF-Q score. However, participants who had been practicing trail running more than 3 years were more likely to screen positive for low energy availability, although this difference did not reach significance (p > 0.050, Cramér’s V = 0.12).

Table 3 presents the medical and gynecological-obstetrical characteristics of the trail runners, as well as the PHQ-9 score. Female trail runners who reported a decrease in libido during training had greater risk of low energy availability (p = 0.043, Cramér’s V = 0.15). No significant differences were found for past pregnancies or births, the use of oral contraceptives, urinary incontinence, pelvic prolapse, or stress fractures. However, participants who screened positive for depression on the PHQ-9 had a greater risk of having positive scores for both the BEDA-Q (p < 0.001, Cramér’s V = 0.28) and the LEAF-Q (p = 0.027, Cramér’s V = 0.13).

**Fig 1:**
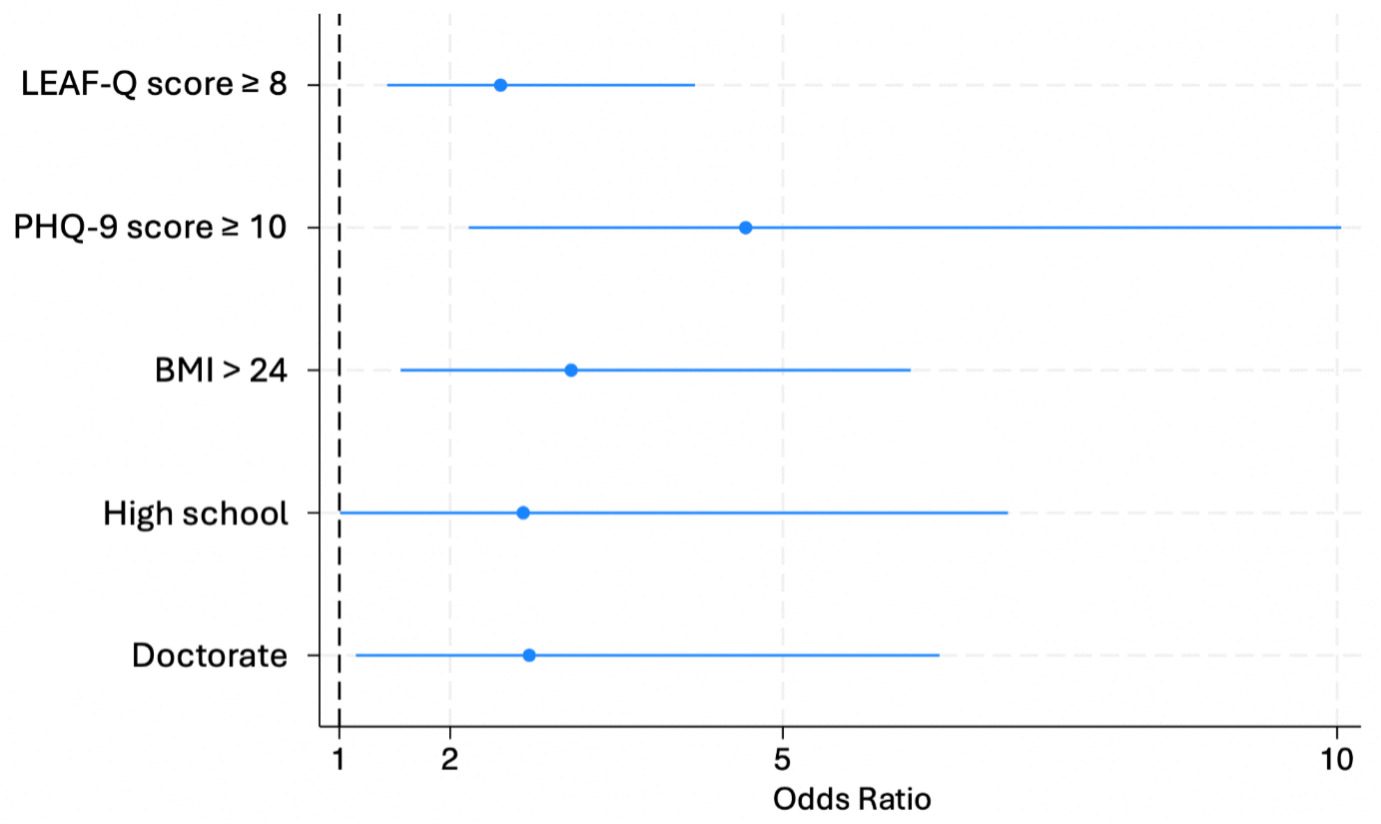
Factors associated with BEDA-Q ≥ 0.27 (N = 276).

Considering all variables from Tables 1 to 3, except height and weight, the multivariate regression analysis for the BEDA-Q (Fig 1) indicated that a positive LEAF-Q score (p = 0.001, OR 2.45, 95%CI 1.43–4.21), a positive PHQ-9 score (p < 0.001, OR 4.67, 95%CI 2.17–10.03), a BMI greater than 24 kg/m^2^ (p = 0.001, OR 3.09, 95%CI 1.55–6.16), and having either a high school (p = 0.049, OR 2.66, 95%CI 1.01–7.03) or a doctoral (p = 0.023, OR 2.71, 95%CI 1.15–6.41) level of education were the strongest associated independent factors. The final multivariate model for the BEDA-Q retained five predictors and given the 148 positive screenings, the resulting EPV ratio was 29.6.

**Fig 2:**
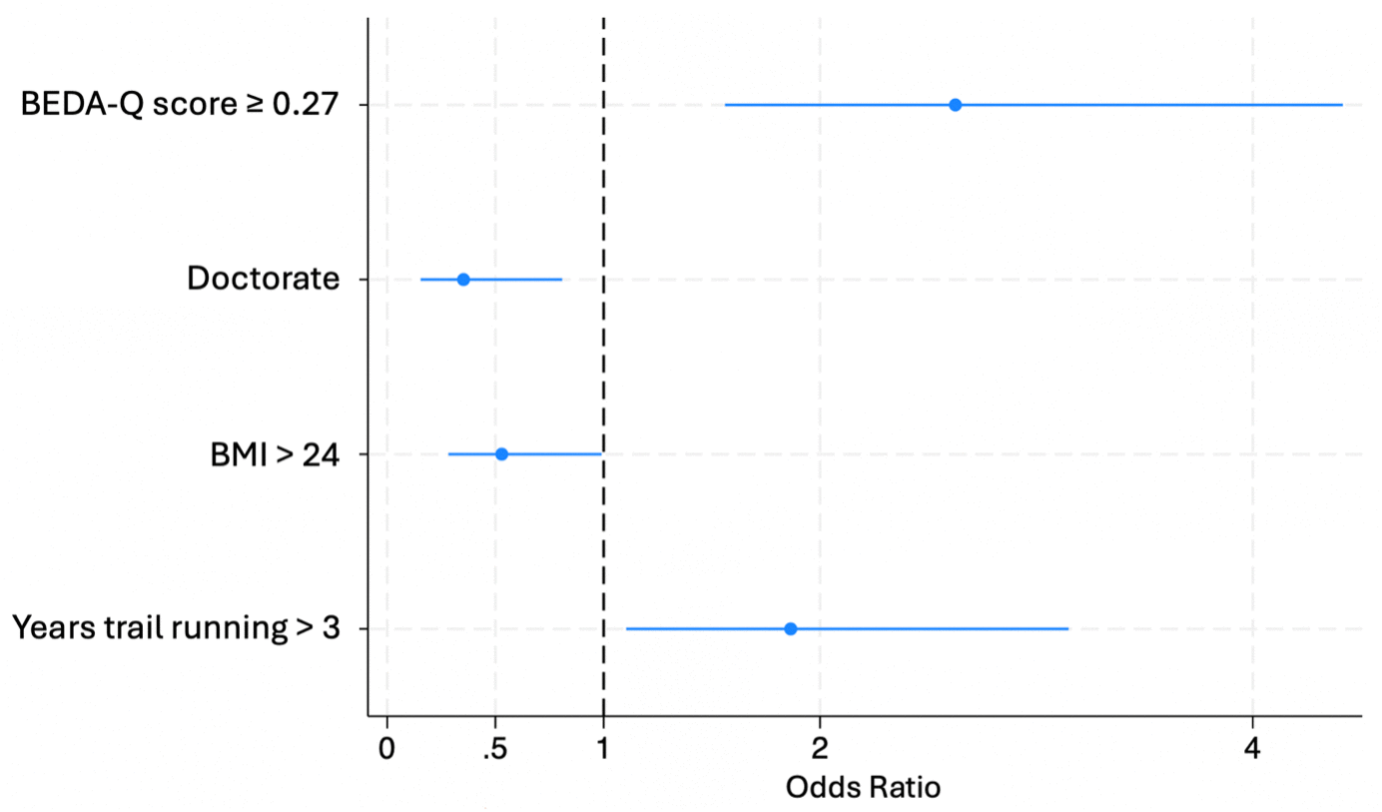
Factors associated with LEAF-Q ≥ 8 (N = 276).

For the LEAF-Q (Fig 2), a positive BEDA-Q score (p < 0.001, OR 2.63, 95%CI 1.56–4.42) and having practiced trail running more than 3 years (p = 0.020, OR 1.86, 95%CI 1.10–3.15) were independent factors positively associated with a LEAF-Q score above 8, whereas a BMI greater than 24 kg/m^2^ (p = 0.047, OR 0.53, 95%CI 0.28–0.99) and a doctoral level of education (p = 0.014, OR 0.35, 95%CI 0.15–0.81) were inversely associated with a positive LEAF-Q score. The final multivariate model for the LEAF-Q retained four predictors and with 152 positive screenings, the model achieved an EPV ratio of 38.0.

## Discussion

This study aimed to investigate the occurrence of EDs and LEA, and to identify associated factors among female trail runners, contributing to the limited literature focusing on women in this sport.

Results showed that 53.6% of female trail runners screening positive for EDs, consistent with prior research reporting a 48% prevalence rate in this population, despite using a different screening instrument [35]. These rates are notably higher than those observed in other female endurance athletes, where the prevalence of EDs ranges from 21% to 32% in marathon [4], ultra-marathon [10] and broader endurance sports athletes [9,11]. The prevalence of LEA risk among female trail runners was 55.1%, which is comparable to findings from previous studies in female endurance runners [9,10,24,26,28], including a study on trail runners reporting a prevalence of 49.7% [35].

The independent risk factors associated with a positive BEDA-Q score were BMI, education level, and PHQ-9 scores. Participants with a BMI above 24 kg/m^2^ had three times greater odds of screening positive for EDs than those with a lower BMI. This positive association between BMI and EDs has been demonstrated in other female athlete populations [9,10,43]. This can be explained by the tendency of women with higher body weight to more likely report lower satisfaction with their appearance and engage in restrictive eating behaviours [44], especially in sports where leanness is seen as an advantage [3,4]. However, a lower BMI might have been expected to be associated with EDs due to restrictive energy intake. In the context of trail running, this finding may reflect the greater importance of muscle mass for uphill and downhill performance, which could contribute to a higher BMI despite the presence of disordered eating behaviours. Interestingly, unlike the results observed with the BEDA-Q, a BMI above 24 kg/m^2^ was associated with a reduced risk of LEA. This negative correlation makes physiological sense, as individuals with higher BMI, especially higher fat mass, may have greater energy stores and may therefore be less likely to develop the chronic negative energy balance that defines LEA [21]. Although some studies have also demonstrated this negative association in female athlete populations [9,45], other studies show no difference in BMI comparing female endurance athletes at risk of LEA [21,24,25,27,28].

Education level was also found to be significantly associated with both BEDA-Q and LEAF-Q scores. Having only a high school education and holding a doctoral degree were both associated with more than a twofold increase in the risk of EDs. Research examining the association between educational attainment and EDs prevalence has produced mixed results, with some studies finding links to both higher and lower education levels, while many show no clear relationship [46,47]. This inconsistency may explain the varied findings observed in our study. On the other hand, having a doctoral degree appeared to be a protective factor against LEA. A study found that athletes with the greatest awareness of the Female Athlete Triad had primarily gained their knowledge through school or university education [10]. This suggests that higher levels of education may contribute to increased awareness of RED-S and its consequences, and could potentially serve as a protective factor against the development of the syndrome. This highlights the importance of implementing educational interventions as a key strategy for prevention.

Additionally, a positive screening for depression was linked to an almost five times greater risk for EDs. This finding likely reflects the high prevalence of depression among individuals with EDs [48,49]. A positive PHQ-9 score was also found to be associated with a higher risk of LEA among female trail runners. This is expected, as impaired mental health, including depressive symptoms, is recognized as an important health outcome of RED-S [50], reinforcing the importance of early identification of those at risk of these conditions.

While previous research links high training loads and frequency with an increased risk of LEA in athletes [25,51,52], our study did not find such associations. Instead, we observed that the total years practicing trail running was independently associated with the LEA risk. Female trail runners who practiced the sport more than three years were nearly twice as likely to screen positive on the LEAF-Q. This suggests that the cumulative duration of engagement in the sport, rather than current training intensity, may contribute to prolonged exposure to energy imbalance and subsequent disturbances. An alternative explanation is the theory of allostatic load, which refers to the cumulative physiological and psychological “wear and tear” resulting from chronic exposure to stress [53]. From this perspective, symptoms commonly attributed to LEA, including menstrual disturbances, impaired immunity, and depressive symptoms, may emerge not solely from insufficient energy intake, but from the combined burden of training stress, life stressors, and psychosocial pressures. Chronic activation of stress-response systems contributes to hormonal disruption and mood disturbances that overlap with LEA-related symptom profiles [29]. Thus, mental health symptoms and other clinical manifestations observed in athletes could reflect a broader stress-adaptation imbalance rather than energy deficiency alone.

Furthermore, we found that women reporting a decrease in libido during training periods were more likely to screen positive for LEA. An association between intensive endurance training and reduced libido has previously been demonstrated in male athletes [52,54,55], where high training loads are linked to decreased testosterone levels and diminished sexual drive. Although this relationship remains understudied in female athletes, a similar mechanism may be present, just as LEA disrupts female sex hormones and menstrual function, it may also result in alterations in libido. This emphasizes the importance of expanding research into gynaecological health in sport.

Many studies have reported that younger athletes are more likely to screen positive for EDs [26,35] and LEA [24,26,35]. However, our findings did not reflect this trend, which may be explained by the age cutoffs we used. While previous research often identifies significant differences within the 18–24 age group, our “younger” category extended up to 30 years old, potentially masking age-related effects. This interpretation is supported by clinical studies showing that women who are less than 14 years past menarche are more sensitive to fluctuations in sex hormones associated to LEA compared to older females [56].

Finally, in the present study, 34% of female trail runners were identified as being at risk for both EDs and LEA. Athletes who screened positive for LEA were more than twice as likely to also screen positive for ED, and conversely, those who screened positive for EDs had over a twofold increased likelihood of screening positive for LEA. This statistical association is consistent with previous findings in athletic populations, particularly among female endurance athletes, where EDs and LEA frequently coexist [9,10,26,28,35]. EDs are well-established risk factors for LEA [57–60], a relationship supported by our findings, as 61.8% of runners identified as at risk for LEA also screened positive for EDs. This proportion is notably higher than that reported in other studies of female endurance athletes [9,21], which may suggest a stronger association between EDs and LEA in female trail runners. One possible explanation is the specific demands of trail running, which involves prolonged training durations, substantial elevation gain, and high cumulative energy expenditure, making adequate fuelling particularly challenging. In this context, restrictive eating behaviours may more readily lead to sustained energy deficits. Additionally, trail running often values leanness, particularly for uphill performance [61], which may reinforce the belief that lower body weight improves performance and consequently lead to more weight-control behaviours and amplify the impact of disordered eating on energy availability. At the same time, performance in trail running may also depend significantly on muscle strength, which could explain the presence of higher BMI despite eating disorders. Finally, the logistical challenges of trail running, including long training sessions in remote environments, may further limit opportunities for adequate fuelling, thereby strengthening the link between EDs and LEA in this population. It is nevertheless important to consider the potential conceptual overlap between the BEDA-Q and the LEAF-Q. Although these instruments are designed to screen for distinct conditions, they may capture different dimensions of the same underlying energy-related disturbance. Consequently, the statistical association between these tools likely reflects an overlap in behavioural and physiological manifestations of a shared energy-deficient state. As both instruments are high-sensitivity screening tools, their symptom profiles may partially overlap, which could contribute to the strength of the observed associations. Nonetheless, the elevated rates of EDs and LEA highlight the importance of implementing prevention and intervention strategies for female trail runners.

Prevention strategies, such as screening and educational interventions, are considered the most effective to reduce the health and performance consequences of EDs and LEA [62–64]. Given that awareness and understanding of EDs and LEA remain limited among female athletes and their coaches [10,50], educational interventions targeting athletes, coaches, healthcare providers, and support networks are vital to inform about the detrimental physiological and performance consequences of these conditions. Understanding key factors such as menstrual health, injury history, and eating behaviours is crucial for coaches and practitioners to identify early signs of EDs or LEA and intervene appropriately [35]. Coaches should routinely inquire about these aspects before prescribing demanding training loads to ensure athletes’ needs are met and risks minimized.

The present study has limitations. First, it relied on anonymous self-reported data and responses, and clinical diagnoses could not be verified. Second, the BEDA-Q and the LEAF-Q are useful for screening purposes, but they are not diagnostic tools and additional evaluation is necessary for accurate assessment and effective intervention [15,21,65]. Furthermore, it is important to acknowledge that the RED-S model remains a conceptual framework rather than a universally recognized clinical syndrome, and the symptoms identified via these screenings may reflect a broader psychophysiological imbalance rather than a definitive diagnosis of energy deficiency [29]. The predictive model was based primarily on individual characteristics, training variables, and medical factors. This limits consideration of broader cultural, social, and normative influences on athletes’ body image. Future studies could adopt multi-method assessments to improve validation and identify additional factors potentially associated with EDs and LEA, however, such approaches may reduce feasibility in large populations. Moreover, the cross-sectional design of this study limits our ability to establish causal relationships between the variables. Consequently, the findings should be interpreted as associations that may reflect reciprocal influences rather than a causal pathway. Future studies should focus on the long-term gynecological health of these athletes, including aspects such as changes in libido, which remain underinvestigated and were found to be significant in our study. Finally, several continuous variables were dichotomized to improve practical interpretability for sports practitioners. However, we acknowledge that this may have limited our ability to detect more subtle dose-response relationships and may have reduced overall statistical power.

To our knowledge this is the first study to investigate EDs and LEA specifically among female trail runners. Key independent risk factors for EDs included BMI above 24 kg/m^2^, depression and education level. For LEA, practicing trail running more than three years was the sole independent risk factor identified, while BMI above 24 kg/m^2^ and having a doctoral degree were found to be protective factors. Additionally, we demonstrated a reciprocal statistical association between EDs and LEA within this population. Given the elevated risk of these conditions among female trail runners and their serious health consequences, implementing educational prevention strategies and regular screening is essential to protect the health and well-being of these athletes.
